# Beginning of the end of Onchocerciasis in the Americas

**Published:** 2013-09-30

**Authors:** Gloria I. Palma, Sofía Duque, Ruben Santiago Nicholls

**Affiliations:** 1 Programa Nacional de Eliminación de la Oncocercosis. Departamento de Microbiología, Universidad del Valle, Cali ,Colombia.; 2 Programa Nacional de Eliminación de la Oncocercosis. Grupo de Parasitología Instituto Nacional de Salud, Bogotá DC, Colombia; 3 Instituto Nacional de Salud, Bogotá DC, Colombia

Onchocerciasis, also known as River Blindness, is a parasitic disease caused by the nematode *Onchocerca volvulus* and transmitted by black flies of the genus *Simulium. *It is endemic in Africa, where an estimated 37 million people are infected. It is almost certain that the slave trade in the 17^th^ and 18^th^ centuries brought onchocerciasis from West Africa to the Americas^1^, where transmission foci where established in six countries: Mexico, Guatemala, Venezuela, Brazil, Ecuador and Colombia. Since the beginning of the 20^th^ century it was suspected that this vector borne disease was present in Colombia but the first confirmed case was not reported until 1965. The exact location of the single focus in the country was confirmed almost thirty years later in locality of Naicioná, on the stream that bears the same name^2^. 

Initial epidemiological/ parasitological surveys were carried out by researchers from the Universidad del Valle- Tulane University ICMRT (International Center for Medical Research and Training) between 1965 and 1970 and from the Universidad del Valle Department of Microbiology and CIDEIM (Centro Internacional de Investigaciones y Entrenamiento Medico) in 1977 and 1989 ^3^. 

 In September 1993, the National Onchocerciasis Committee was established in response to PAHO's Directing Council Resolution CD35R.14 and to advocacy efforts by OEPA (Onchocerciasis Elimination Program of the Americas). A multidisciplinary, multi-institutional team was assembled to carry out a thorough active search for infection in southwestern Colombia. The team composed of researchers from the National Institute of Health of Colombia, the National University of Colombia, the Hospital VozAndes of Ecuador and the Universidad del Valle, confirmed the existence of a single onchocerciasis focus in Colombia located, not on the Micay River as previously thought, but on a stream that flows into one of its tributaries, the Chuare River while ruling out the existence of a transmission focus in the border between Colombia and Ecuador^2^. 

Once the baseline studies were carried out, the National Onchocerciasis Elimination Program, lead by the National Institute of Health of Colombia, with technical and financial support from Onchocerciasis Elimination Program for the Americas (OEPA), began the mass drug administration (MDA) of ivermectin (Mectizan®) donated by Merck through the Mectizan Donation Program. Ivermectin is an anti-helminth drug that kills the disease producing *O. volvulus* microfilariae in the skin and eyes and renders adult females infertile for at least 6 months. The basic strategy for achieving elimination is through MDA of ivermectin twice a year to at least 85% of all the eligible population for at least 10 consecutive years. The Colombian MDA ivermectin program was begun in September 1996 as a community- based program complemented by social mobilization and the promotion of community participation and ended in November 2007. During these eleven years, 23 treatment rounds were carried out. Coverage of at least 85% of the eligible population was achieved in 19 consecutive rounds, beginning with the 5th one through the 23rd and last one. (Nicholls *et al*, unpublished results). This was made possible through the dedicated work of community health workers who were trained for distributing ivermectin to all the elegible population and to the acceptance of the periodic treatments by the community. Maintaining the interest of the community throughout all these years was the result of a continued effort to carry out health education, community participation and social mobilization programs which included onchocerciasis but went beyond to address other health concerns of the inhabitants of Naiciona such as hypertension, nutrition, soil-transmitted helminths and intestinal parasites, food security, alcoholism. Other issues related with their living conditions such as literacy, education and conflict prevention and resolution were also addressed.

Beginning January 2008 a Post-Treatment Surveillance (PTS), also known as the Pre-certification period was begun. This is defined by WHO as "the period following interruption of transmission during which surveillance is carried out to verify that interruption of transmission is sustained after ceasing all control interventions"^4^. The 3 year PTS was carried out between January 1, 2008 and December 31, 2010. Frequent contact, at least 4 times a year, was kept with the people living in Naiciona . Activities such as health education, health promotion and prevention, nutrition, food security, social mobilization and annual updating of the census were carried out during this time. An new entomological assessment was carried out at the end of this period. All the 13,481 female black flies studied were negative for *O. volvulus*, thus confirming the elimination of onchocerciasis transmission in the Colombian focus.

 In November 2011, the Ministry of Health and Protection presented a formal request to WHO for certification of the elimination of the Director General of WHO formally announced in April 2013 that Colombia was the first country in the Americas and the world to have been verified by WHO as having eliminated the transmission of onchocerciasis.

The Onchocerciasis Elimination Program of Colombia was a joint venture of researchers in two universities with Colombian public health entities like the National Institute of Health, the Health Service of the Department of Cauca and the Ministry of Health during more twenty years. It also provided an opportunity for the community of Naiciona to receive attention from the local, state and national authorities in order to improve its living conditions. Otherwise, Naiciona would most likely still be one amongst thousands of remote villages along the Colombian Pacific Coast whose inhabitants have long been neglected, living in very poor socioeconomic and environmental conditions, with important access barriers to health services, education, proper drinking water and basic sanitation. 

It can now be said with certainty that, for the first time in at least 300 years, and hopefully forever, the territory occupied by modern day Colombia is free from the risk of onchocerciasis disease and transmission. Though onchocerciasis in Colombia was circumscribed to a small focus in a remote village, this is an important public health milestone both for the country as well as for the Americas region. It is proof of principle that ambitious long-term public health goals can be attained as long as the programs have the adequate political, financial and technical support. The lessons learned will certainly be useful for advancing towards the reduction of the disease burden caused by other neglected tropical diseases in Colombia and for the achievement of goals such as the elimination of vector-borne domestic transmission of Chagas disease, the elimination of blinding trachoma and the control of soil-transmitted helminth infections. 
